# Fatal West Nile Virus Infection in Horse Returning to United Kingdom from Spain, 2022

**DOI:** 10.3201/eid3002.230690

**Published:** 2024-02

**Authors:** Mirjam Schilling, Bettina Dunkel, Tobias Floyd, Daniel Hicks, Alex Nunez, Falko Steinbach, Arran J. Folly, Nicholas Johnson

**Affiliations:** Animal and Plant Health Agency, Addlestone, UK (M. Schilling, T. Floyd, D. Hicks, A. Nunez, F. Steinbach, A.J. Folly, N. Johnson);; Royal Veterinary College, Hatfield, UK (B. Dunkel);; University of Surrey School of Veterinary Medicine, Guildford, UK (F. Steinbach)

**Keywords:** West Nile virus, WNV, zoonoses, equine, vector-borne infections, flavivirus, viruses, Spain, United Kingdom

## Abstract

We report fatal West Nile virus (WNV) infection in a 7-year-old mare returning to the United Kingdom from Spain. Case timeline and clustering of virus sequence with recent WNV isolates suggest that transmission occurred in Andalusía, Spain. Our findings highlight the importance of vaccination for horses traveling to WNV-endemic regions.

A West Nile virus (WNV) outbreak among equids occurred in Andalusia, Spain, in 2020, and subsequent cases were reported in 2021 and 2022 (*1*). Thus far, WNV originating in the United Kingdom has not been detected, and surveillance of birds is used to monitor for possible introduction. WNV was previously detected in the United Kingdom in a horse returning from Cyprus, where WNV circulates seasonally (*2*). Serologic data suggest that <30% of horses in the United Kingdom are currently vaccinated because WNV is not endemic (*3*). The risk factors that predispose horses to developing neurologic disease after WNV infection are unknown. 

In November 2022, a 7-year-old mare showing clinical signs of ataxia, hyperesthesia, and reluctance to move was admitted to the Royal Veterinary College Referral Hospital (Hatfield, UK). The mare had just returned to the United Kingdom after 1 month in Andalusia and 2 days of traveling through France. Quantitative reverse transcription PCR (qRT-PCR) performed on a nasopharyngeal swab sample was negative for equine herpesviruses 1 and 4. Because of the mare’s travel history and seasonal presence of WNV in Spain, serum was submitted to the Animal and Plant Health Agency for WNV antibody testing. The result of ELISA testing for detection of WNV IgM (IDvet, https://www.innovative-diagnostics.com), indicative of recent infection or vaccination, was positive. The mare had no record of having received WNV vaccine. WNV-specific qRT-PCR (*4*) failed to detect virus in serum and cerebrospinal fluid samples. After receipt of anti-inflammatories (0.1 mg/kg dexamethasone), the mare’s condition improved initially, allowing her discharge from hospital. However, after discharge, her condition deteriorated and she was readmitted to the referral hospital, where she was euthanized because of rapid progression of neurologic signs leading to recumbency.

A full postmortem examination performed under Biosafety Level 3 containment showed no substantial gross pathology. Histopathologic investigation of the central nervous system (CNS) revealed mild nonsuppurative inflammation, affecting predominantly gray matter of the brainstem and spinal cord, consistent with a viral infection. Microscopic examination of peripheral nervous tissue and a range of viscera (heart, skeletal muscle, lung, liver, kidney, spleen, and submandibular lymph node) revealed no substantial pathologic changes. Immunohistochemical staining with Kunjin virus primary antibodies (nonstructural 1, rabbit; Australian Animal Health Laboratory, https://www.csiro.au/en/about/facilities-collections/acdp**)** detected small amounts of virus antigen in association with inflammatory foci in the spinal cord only. No specific immunostaining was found elsewhere in the CNS or any other tissues. WNV-specific qRT-PCR detected WNV RNA in brain tissue (mesencephalon, cerebral cortex, and medulla oblongata) and in the spinal cord. A partial genomic sequence was obtained from RNA samples of the spinal cord by next-generation sequencing. The resulting phylogeny showed clustering of the retrieved virus sequence with recent isolates of lineage 1a from mosquitoes and humans in Andalusia (Figure 1) (*5*), suggesting that transmission most likely occurred in Andalusia.

**Figure 1:**
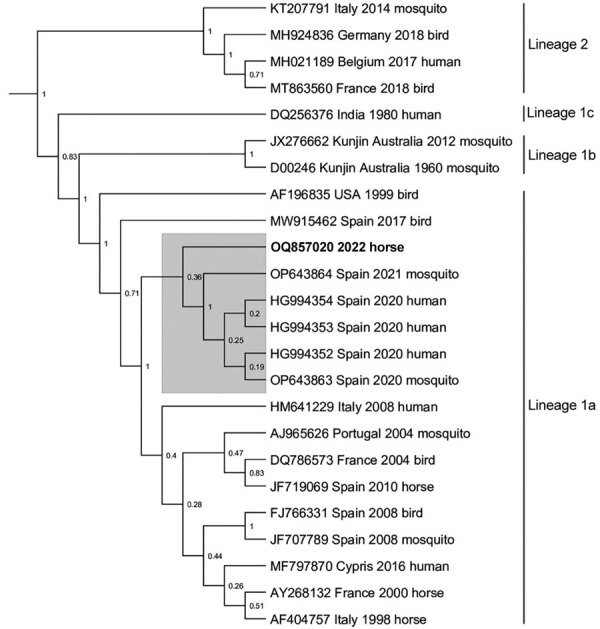
Next-generation sequencing data associated with fatal West Nile virus (WNV) infection in a horse returning to the United Kingdom from Spain, 2022. Bayesian phylogenetic tree analysis of a 624-bp sequence located in the nonstructural 5 gene showed that the strain from the horse (boldface; GenBank accession no. OQ857020) clusters with recent sequences from Andalusia, Spain (gray shading). Next-generation sequencing was conducted on an Illumina MiSeq sequencer (https://www.illumina.com). The sequence was aligned with 23 WNV lineage 1 and 2 reference sequences obtained from GenBank in MEGA version 11.0.13 (https://www.megasoftware.net), and a Bayesian phylogenetic analysis was undertaken in BEAST version 1.10.4 (https://beast.community) using a general time reversible plus invariant sites plus gamma nucleotide substitution model and 10,000,000 Markov chain Monte Carlo generations. Node labels represent posterior probabilities. Accession number, country, year of detection and host species are included for each sequence.

In conclusion, travel history, clinical examination, laboratory testing, and postmortem examination combined indicated an acute WNV infection in this horse (Figure 2). In contrast to cases in birds and some humans, WNV infection in horses seems to be characterized by encephalitic lesions with little associated antigen, occasionally even a discrepancy between distribution of virus antigen and location of lesions (*6*). Although IgM is detectable 4–6 weeks after infection, the low levels of virus in CSF, blood, and tissue are consistent with earlier descriptions of WNV infections in horses (*7*).

**Figure 2:**
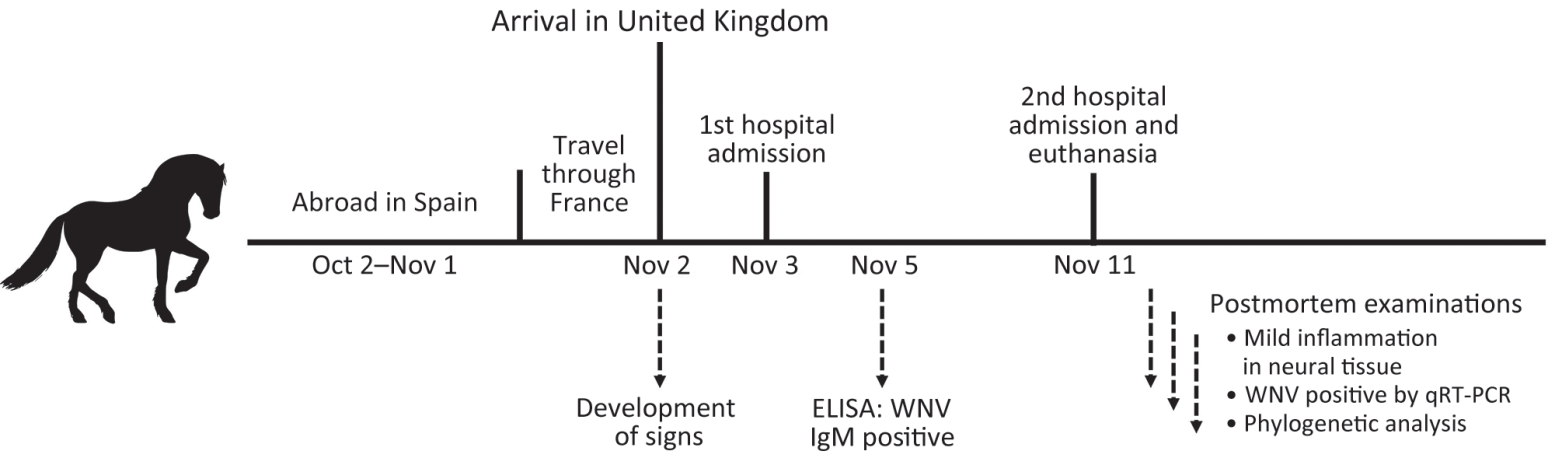
Timeline of events associated with fatal WNV infection in horse returning to the United Kingdom from Spain, 2022. qRT-PCR, quantitative reverse transcription PCR; WNV, West Nile virus.

Vaccination is strongly advised for all horses traveling to areas where WNV is known to circulate because treatment consists only of symptomatic support, whereas licensed vaccines providing effective protection are available (*8*). In light of the ongoing spread of WNV across the world, an increasing amount of traveling horses are at risk. Veterinary and public health bodies should therefore increase vigilance for this emerging disease and prepare for more WNV infections in the future.
